# Gabapentinoids and unexplained death in general practice: Case series and feasibility study arising from a critical incident

**DOI:** 10.1080/13814788.2026.2645473

**Published:** 2026-03-23

**Authors:** Augustin Gabriel, Tom Fahey

**Affiliations:** Department of General Practice, RCSI University of Medicine and Health Sciences, Ireland

**Keywords:** Gabapentin, Pregabalin, drug-related side effects and adverse reactions, mortality, primary health care

## Abstract

**Background:**

Prescribing epidemiology in general practice shows gabapentinoid drugs to be independently associated with unexpected, drug-related death. There is an increasing trend of gabapentinoid deaths throughout Europe and North America.

**Objectives:**

The overall aim of this study was to assess how patient, practice and health system factors might be associated with gabapentinoid prescribing in primary care.

**Methods:**

Case series following a critical incident of an unexpected death in a patient prescribed a gabapentinoid drug in a single general practice. Unexpected and expected deaths in patients prescribed a gabapentinoid drug deaths over an 11-year period in a single general practice. We examined patient, prescriber and health system factors. Toxicology and post-mortem data were provided by the Coroner.

**Results:**

There were 36 deaths (four unexpected and 32 expected deaths) during the study period. Of the four patients who suffered an unexpected death, one of these patients’ cause of death could be attributed to drug and alcohol toxicity. Over half of gabapentinoid prescribing (*n* = 19,53%) was hospital initiated, often ‘off-label’ (*n* = 6, 17%) and commonly co-prescribed with opiates (*n* = 15, 42%) and benzodiazepines (*n* = 11, 31%) to patients with high multi-morbidity.

**Conclusions:**

Gabapentinoids are often initiated in the outpatient setting in clinically complex patients, often for ‘off label’ indications, with high polypharmacy. Patient, practice and health-system related factors need to be addressed in relation to gabapentinoid associated deaths and reflected in clinical practice guidelines. There is critical value in using toxicology reports from Coroner’s offices in cases of unexplained gabapentinoid death in general practice.

## Introduction

There is an increasing incidence of gabapentinoid (pregabalin and gabapentin) prescribing worldwide, with increased volume of prescribing of these drugs reported in the US, UK, Scotland and Ireland [1–4]. Alongside the increased volume of gabapentinoid prescribing is co-prescribing with other psychotropic and pain medication such as opiates, benzodiazepine and z-class drugs [5] Evidence is accumulating that gabapentinoid prescribing alone and when co-prescribed are independently associated with increases in drug-related deaths, with risk of death increasing with stronger doses of opiates and/or gabapentinoids [6–8].

This emerging evidence has prompted national regulatory bodies to take action in terms of issuing risk warnings to the public and to prescribing physicians. For instance, the Federal Drug Agency (FDA) has warned of serious breathing difficulties with gabapentin and pregabalin use in patients with existing respiratory risk conditions and/or respiratory risk factors. Respiratory risks include: use of central nervous system (CNS) depressants or opioids; chronic respiratory conditions such as chronic obstructive pulmonary disease (COPD) as well as demographic risk factors, particularly older age [9]. The evidence brought forth by the FDA was based on forty nine case reports between 2012 to 2017 where significant adverse events occurred, including drug-related death [9].

Whilst the risk of gabapentinoids are becoming clearer, the underlying reasons behind the initial initiation of the drug is frequently less clear. Patient, physician and health system factors all play a role in the increase in prescribing of gabapentinoids [1,10]. The public health crisis of opiate prescribing, particularly in the US, has brought into focus that effective strategies require a multifactorial understanding at the patient, physician and health system levels. Though general practitioners prescribe the greatest volume of opiates, there is evidence that hospital initiation of opiates in opiate-naïve patients at discharge is increasing and may be an important driver of longer-term opiate use [11]. Whether the same issue arises in the case of gabapentinoid prescribing is not yet known.

This case series was prompted by a critical incident in a single general practice. Critical incident reporting is an effective way to address patient safety in a general practice and enable a culture of quality improvement [12]. A female patient in her 60’s taking long-term gabapentin was found dead at home. She was well known to the general practitioners who had been prescribing painkillers for a variety of pain-related complaints. It was acknowledged by the medical team and discussed with the patient that the combination of Gabapentin, Tapentadol and Zoplicone significantly increased her risk for drug related toxicity. It had proved very difficult to modify this patient’s drug regimen, particularly as initiation of these drugs has taken place in a hospital-based pain clinic.

The primary aim of this case series, prompted by a critical incident of a drug-related death, is to investigate how patient, physician and health-system factors influence gabapentinoid prescribing in primary care, and to assess their contribution to unexplained deaths. The objectives were to: quantify gabapentinoid prescribing over a 11-year period; examine the cause of death in patients taking gabapentinoid drugs at the time of their death for both expected and unexpected deaths using post mortem and toxicology reports when unanticipated deaths occurred. Lastly, this study in a single general practice served as a feasibility study to a case control study that would examine modifiable and non-modifiable factors that might reduce the likelihood of gabapentinoid associated deaths in a larger, more representative sample of general practices.

## Methods

In this case series, we examined gabapentinoid prescribing in a single general practice over a 11-year period. We extracted the following variables from each patient’s clinical record(1) patient characteristics: age, gender, co-morbidity, co-prescription of drugs implicated in drug-related death (benzodiazepine, z-class, opiate and alcohol), and the clinical indication for initiation of gabapentinoid prescription; (2) physician related prescribing issues- the clinical setting of medication initiation (general practice or hospital clinic) and specialty of the prescribing physician; and (3) health system factors in terms of monitoring or changes in prescriptions.

For patients who had died, we classified the deaths as expected where a known terminal condition had been diagnosed and palliative care initiated. Unexpected death occurred when a patient died suddenly and their death had not been expected by their general practitioner or other attending health professionals. For unexpected deaths we wrote to the Coroner requesting post mortem details and toxicology results (blood and urine).

Prompted by an unexpected death, this critical incident study was carried out in a practice where one of the authors (TF) is a general practitioner for the past 18 years. AG collected and prepared the practice data as part of a research summer student placement based in the Department of General Practice, RCSI University of Medicine and Health Sciences in 2023.

## Results

### Descriptive statistics of gabapentinoid prescribing

**Figure 1.**
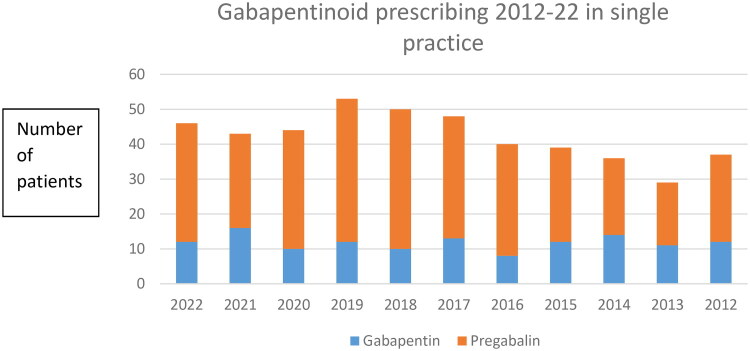
Gabapentinoid prescribing 2012–22 in single practice.

The time trends of prescribing according to type of gabapentinoid drug is summarised in Figure 1. Prescribing peaked in 2019 and reduced slightly in the following years. Similar to national data, females accounted for approximately three quarters of individuals prescribed gabapentinoids and pregabalin was prescribed in two thirds of individuals during the 2012–22 time period.

### Gabapentinoid deaths- unexpected and expected

There were 36 deaths (four unexpected and 32 expected) during the study period. Table 1 provides details of patients who died and were concurrently prescribed a gabapentinoid at the time of their death. The majority of patients were over the age of 70 (*n* = 29, 81%) and female (*n* = 19, 53%). Patients also had significantly high multimorbidity (median Charlston Index score 7, range 1 to11) and polypharmacy (median number of drugs 11, range 2 to 21) scores.

**Table 1.** Patient characteristics- expected* and unexpected deaths.

ID	Age	Sex	Charlston Comorbidity index	Number of medicines co-prescribed	Gabapentinoid Prescribed	Clinical Indication for Gabapentinoid	Specialty Hospital Initiated	Off Label	Opiate	Benzodiazepine	Alcohol misuse	Principal cause of death
1*	60–69	F	11	21	Gabapentin 100 mg daily	Low back pain	Pain Yes	No	Tapentadol 100 mg BD	Zopiclone 7.5 mg	Yes	Mixed drug and alcohol toxicity
2*	50–59	F	10	10	Pregabalin 100 mg QDS	Sciatica	Pain Yes	No	Oxycontin 10 mg Oxynorm 5 mg	Alprazolam 1 mg	No	Rupture Aneurysm
3*	80–89	F	11	10	Pregabalin 150 mg daily	Dermato myositis	Rheumatology Yes	Yes	No	No	No	Cardiac arrhythmia; IHD
4*	70–79	F	3	12	Gabapentin 600 mg BD	SLE	Rheumatology Yes	Yes	No	Zolpidem 10 mg	No	Cardiac arrhythmia; IHD
5	70–79	F	9	14	Gabapentin 300 mg BD	Unclear	Unclear	Unclear	No	Zopiclone 10 mg	No	Metastatic Breast Cancer
6	70–79	M	10	2	Gabapentin 100 mg BD	Unclear	Unclear	Unclear	No	No	No	Old age
7	90–99	M	6	8	Gabapentin 100 mg TDS	Sciatica	Pain Yes	No	No	Zolpidem 10 mg	No	Metastatic Colorectal Carcinoma
8	80–89	F	8	13	Gabapentin 300 mg TDS	Tempero-Mandibular Pain	Pain Yes	Yes	No	Zopiclone 7.5 mg	No	COPD
9	30–39	F	2	8	Gabapentin 100 mg TDS	Neuropathic cancer pain	Oncology Yes	No	Tramadol 100 mg QDS	No	No	Metastatic Cancer
10	80–89	M	7	20	Gabapentin 100 mg	Unclear	Geriatrics Yes	Unclear	No	No	No	CVA
11	80–89	M	7	11	Gabapentin 100 mg TDS	Peripheral Neuropathy diabetic	Diabetes Yes	No	No	No	No	IHD
12	80–89	M	7	13	Gabapentin 100 mg BD	Unclear	Unclear	Unclear	No	No	No	Intersitital Lung Disease
13	70–79	M	5	9	Gabapentin 100 mg TDS	Unclear	Unclear	Unclear	Tadenafil 100 mg daily	No	No	Lung Cancer
14	80–89	M	11	15	Gabapentin 100 mg TDS	Unclear	Unclear	Unclear	Oromorph	Zolpidem 10 mg	No	Prostate Cancer
15	70–79	F	11	15	Gabapentin 100 mg BD	Unclear	Unclear	Unclear	Morphine Sulphate (MST)	No	No	Metastatic Renal Cell Carcinoma
16	80–89	F	5	11	Pregabalin 75 mg daily	Unclear	Unclear	Unclear	No	Alprazolam 0.25 mg BD	No	Respiratory Failure
17	70–79	F	6	11	Pregabalin 75 mg	Sciatica	Pain Yes	No	No	No	No	Lung Cancer
18	60–69	M	4	9	Pregabalin 25 mg Capsules	Ano-Rectal Cancer	Oncology Yes	Yes	Oxycontin	Temazepam 10 mg	No	Lung Cancer
19	80–89	F	5	4	Pregabalin 75 mg daily	Unclear	Unclear	Unclear	No	No	No	Unclear
20	70–79	M	7	13	Pregabalin 75 mg BD	Neurogenic Pain	Oncology Yes	No	Fentanyl Hydromorphine	No	No	Renal Cell Carcinoma
21	90–99	F	7	11	Pregabalin 100 mg	Osteoporosis pain	Rheumatology Yes	Yes	No	No	No	Pancreatic Cancer
22	80–89	F	7	12	Pregabalin 225 mg	Sciatica	Endocrinology Yes	No	Buprenorphine	No	No	Old age
23	30–39	M	2	14	Pregabalin 150 mg BD	Unclear	Unclear	Unclear	Morphine Sulphate	No	No	Stage 4 Non Small Cell Lung Cancer
24	80–89	M	6	20	Pregabalin 125 mg	Unclear	Unclear	Unclear	No	No	No	Old age
25	80–89	F	4	18	Pregabalin 75 mg	Unclear	Unclear	Unclear	Buprenorphine	No	No	Multiple Myeloma
26	70–79	M	10	13	Pregabalin 25 mg	Unclear	Unclear	Unclear	Oxycondone	No	No	Metastatic Bladder Cancer
27	50–59	M	1	14	Pregabalin 225 mg	Neurogenic Pain	Pain Yes	No	No	Chlordiaze poxide	No	Suicide
28	80–89	F	5	12	Pregabalin 75 mg	Unclear	Unclear	unclear	No	No	No	Old age
29	80–89	M	8	13	Pregabalin 75 mg	Neurogenic Pain	Diabetes Yes	No	No	No	No	Dementia
30	80–89	F	5	11	Pregabalin	Unclear	Geriatrics Yes	No	No	No	No	Old age
31	60–69	M	5	8	Pregabalin 75 mg	Unclear	Unclear	Unclear	No	No	No	Gastric Cancer
32	80–89	F	7	8	Pregabalin 50 mg	Unclear	Unclear	Unclear	No	No	No	Sepsis
33	80–89	F	10	13	Pregabalin 75 M mg BD	Unclear	Unclear	Unclear	No	Zopiclone	No	COPD
34	30–39	M	3		Pregabalin 25 mg	Metastatic Cancer	Oncology Yes	Yes	Hydromorphone Oxynorm	No	No	Duodenal Cancer
35	80–89	M	6	13	Pregabalin 25 mg	Sciatica	Pain Yes	Yes	Buprenorphine	No	No	Pneumonia
36	70–79	M	5	10	Pregabalin Lyrica 50MG Capsules	Unclear	Unclear	Unclear	Oxycontin Oxynorm	No	No	Lung Cancer

Hospital initiation of gabapentinoids occurred in 19 (53%) of patients. Off label use was determined in 6 (17%) patients but unclear in a further 16 (44%). Co- prescription of other harmful drugs was common with 15 (42%) taking an opiate, 11 (31%) taking a benzodiazepine; 4 (11%) taking both and 22 (61%) taking either an opiate or benzodiazepine. There was no clinical note entry for harmful alcohol consumption in any of the 36 patients.

Four patients died unexpectedly during the study period. Table 2 provides details of post mortem findings and toxicology results. Patient 1 was found to have their cause of death attributed to drug and alcohol toxicity; this was the patient who prompted the initial critical incident. Two other patients had drug levels in the therapeutic range at the time of their death. Patient 2 had a blood toxicology report that showed benzodiazepine and opiate in the therapeutic range with pregabalin in their urine toxicology. Patient 3 had blood toxicology in the therapeutic range for pregabalin, diphenhydramine and ketamine. Patient 4 had a negative toxicology screen at the time of their death.

**Table 2.** Descriptive characteristics, clinical indication, prescribing history and post mortem and toxicology findings.

Patient 1
Descriptive characteristics- female; aged between 60–69, Charlston co-morbidity index 11; polypharmacy 21 medications; history of alcohol misuse
Clinical indication- low back pain
Prescribing history- hospital initiated pain clinic; co-prescribed Zopiclone
Circumstances- discovered lying in her bathroom; last spoke with neighbour four days previously
Toxicology- blood toxic levels of citalopram, zopiclone; therapeutic levels of tapentadol and gabapentin; ethanol present in blood 91mg%, just below toxic levels (>100mg%). Urine screen positive for citalopram, gabapentin, quinine, tapentadol and zopiclone.
Cause of death- mixed drug and alcohol toxicity
Patient 2
Descriptive characteristics- female; aged between 50–59; Charlston co-morbidity index 10; polypharmacy 10 medications
Clinical indication- low back pain
Prescribing history- hospital initiated pain clinic; co-prescribed Oxycontin
Circumstances- found in bed
Toxicology- blood positive for paracetamol, alprazolam, oxycodone, zolpidem, baclofen, fluoxetine all in the therapeutic range. All present in urine with pregabalin identified additionally. Drugs deemed to be non contributory to the patient’s death
Cause of death- ruptured berry aneurism of the brain
Patient 3
Descriptive characteristics- female; aged between 80–89, Charlston co-morbidity index 11; polypharmacy 10 medications; history of alcohol misuse
Clinical indication- dermatomyositis
Prescribing history- hospital initiated rheumatology clinic; no prescribing of benzodiazepine
Circumstances- fell at home and brought to accident and emergency department where death occurred
Toxicology-blood positive for pregabalin, diphenhydramine, ketamine, paracetamol and propranolol all in the therapeutic range.
Cause of death- hypoxic ischaemic encephalopathy due to presumed cardiac arrhythmia, secondary to ischaemic heart disease
Patient 4
Descriptive characteristics- female; aged between 70 and 79, Charlston co-morbidity index 3; polypharmacy 12 medications
Clinical indication- Systemic lupus erythematosus (SLE)
Prescribing history- hospital initiated rheumatology clinic; no prescribing of benzodiazepine
Circumstances of death- discovered by husband in the morning, in bed; last spoke to husband the previous evening prior to bedtime
Toxicology- blood drug screen negative for ethanol, opiates, cocaine, barbiturates, methadone, cannabinoids, amphetamines, phenothiazines and benzodiazepines
• Cause of death- cardiac arrhythmia secondary to myocardial fibrosis and ischaemic heart disease

## Discussion

### Summary of findings

The epidemiology of prescribing in this single general practice follows a similar pattern as the national prescribing data [8], with an increase in gabapentinoid prescribing during the study period alongside significant co-prescribing with opiates and benzodiazepines, with 22 (61%) of patients taking either drug with a gabapentinoid. Most of the deaths of patients were expected and planned for in terms of a gabapentinoid drug accompanying an opiate drug use for terminal care pain relief. The majority of patients were elderly with significant multimorbidity, receiving polypharmacy drug regimes. Nearly all gabapentinoid prescriptions were initiated in a hospital setting, with a large proportion of patients receiving a gabapentinoid for an ‘off label’ clinical indication.

Of the four unexpected deaths, only one was directly attributable to drug and alcohol toxicity. This confirms the known risks of alcohol with certain psychoactive medications, including gabapentinoids [13]. The three other unexpected deaths were due to other causes with toxicology reports showing combinations of drugs, including gabapentinoids, all within a therapeutic range. These findings demonstrate the value of post mortem and toxicology reports in unexpected community-based deaths when determining true cause of death [14].

The initial critical incident of a drug related death was confirmed at post mortem and toxicology.This case series also shows that many patients take gabapentin in combination with opiates as part of end-of-life care. Only a minority of patients who were prescribed gabapentinoids were at risk of unexpected drug-related death and no other patients were confirmed to have suffered an unexpected drug-related death during this 11-year period. On the one hand, this was a reassuring finding. On the other hand, it highlights the difficulty in combating opiate and/or gabapentinoid-associated death in primary care settings. Each practice will have relatively few ‘at-risk’ patients and any quality improvement initiative such as use of comparative clinical data, audit and feedback and/or use of key opinion prescribing leader will have only limited impact at the individual practice level.

### Comparison with existing literature

There is growing evidence concerning the risk of sudden death with gabapentinoid drugs, particularly in relation to co-prescribing with opiates and/or benzodiazepines [8,15]. This recognition has prompted some drug regulatory authorities to re-classify gabapentinoids, for example the Medicines and Healthcare Regulatory Authority (MHRA) reclassified gabapentins as Schedule II medications and Class C drugs in April 2019 [15]. Our study shows that gabapentinoid related death in this practice was similar in many respects with other community-based deaths, occurring in a patient with known polypharmacy, taking at risk drugs including opiates and benzodiazepines, against a background of ongoing alcohol misuse [6–8].

A recent large community-based randomised controlled trial in the US that used a multi-faceted approach including different forms of communication alongside community-based Naloxone treatment did not have an impact on reducing the incidence of drug-related deaths in primary care [16]. More positively, a recent non-randomised study in hospitalised patients taking a gabapentinoid, a direct-to-consumer (patient) educational brochure had a small but significant impact in deprescribing of the gabapentinoid at 8-week follow up [17].

### Strengths and limitations

This case series shows the value of a critical incident approach after a significant patient safety event, in this case when a patient dies unexpectedly from a drug-related prescribing cause [12].

The limitations of this study are that this is an observational descriptive study limited one single general practice. In order to estimate a practice-based absolute/attributable risk, exposure to all patients from Gabapentinoid drugs during this period would be needed. Some important elements in patient’s clinical records were incomplete or missing: this includes date of initiation and clinical indication for Gabapentinoid; and lastly, blood monitoring data particularly estimated Glomercular Filtration Rate (eGFR) in relation patients with impaired renal function. Our findings require replication in a larger sample of practices, with initial exposure (prescription of gabapentinoid and other drugs) measured at baseline and outcome (drug-related unexpected death) at follow up. More careful characterisation of clinical indication for prescription of gabapentinoid and setting of initiation (type of hospital clinic and more detailed characteristics of prescribing physician) would also be helpful in terms of potential interventions that may mitigate inappropriate use of gabapentinoids in primary care.

### Implications for research and clinical practice

Findings from this study require replication in a larger sample of practices. It appears to be a feasible approach when examining patient, practice and health-system factors that relate to safer gabapentinoid prescribing. We plan to undertake a larger case control study of unexpected gabapentinoid deaths in primary care in relation to hospital initiated prescribing. Based on our finding of 22 (61%) being exposed to a gabapentinoid drug and either an opioid or benzodiazepine, and assuming a differential 70% exposure in cases (unexpected gabapentinoid death) and 50% exposure in controls (prescribed gabapentinoid but alive) we will require 61 cases and 244 controls (ratio of 1:4) to test the hypothesis of a difference in hospital initiation of gabapentinoids between the two groups at 80% power and a significance level of 0.05 (STATCALC, EPI INFO). We plan to recruit between 15 and 20 practices to achieve this sample size. These estimates are based on pragmatic considerations of obtaining four controls for every case of gabapentinoid-associated death and the feasibility of this approach is grounded from this study of four to five unexpected deaths in each general practice.

### Implications for clinical practice

The clinical implications for a single general practice are that care needs to be taken when co-prescribing gabapentinoid drugs with either opiates or benzodiazepines. Much of the prescribing is outside of a general practitioner’s range of initial clinical responsibility; as we have found that gabapentinoids are often hospital initiated (usually from pain, rheumatology or oncology clinics). The challenge for general practitioners is that they have to continue these gabapentinoid prescriptions, monitor co-prescribing and the presence of risk factors that may affect excretion, particularly impaired renal function. General practitioners continue to have clinical responsibility for these hospital-initiated drugs. As was the case with the patient who died unexpectedly, knowledge about the risks of co-prescribing with opiates and/or benzodiazepines needs to be shared with patients, and clearer prescribing protocols between primary and secondary care for gabapentinoid drugs are needed. Unfortunately, current evidence suggests that shifting patients off drugs such as opiates or benzodiazepines is extremely challenging [16,18]. It is likely that discontinuation of gabapentinoids is also likely to be very difficult in primary care without broader psycho-social support.

## Conclusion

Critical incident reporting in terms of prescribing practice is an important element of reflective practice and promotion of a safer prescribing environment in general practice. This study confirms that a large part of gabapentinoid prescribing is hospital initiated [19,20]. General practitioners need to remain vigilant when taking on the responsibility of hospital-initiated drugs, particularly opiates, benzodiazepines, gabapentinoids, particularly when prescribed in combination and for an ‘off-label’ clinical indication.

## Data Availability

Data is available from corresponding author who is the data controller.
